# Assessing salivary matrix metalloproteinase‐8 in prostate cancer patients undergoing androgen deprivation therapy

**DOI:** 10.1002/cre2.624

**Published:** 2022-06-29

**Authors:** Maaz A. Memon, Benish Aleem, Hifza A. Memon, Ka Y. Lee

**Affiliations:** ^1^ Institute of Pathology and Diagnostic Medicine Khyber Medical University Peshawar Pakistan; ^2^ Dow University of Health Sciences Karachi Pakistan; ^3^ Department of Health Sciences, Swedish Winter Sports Research Centre Mid Sweden University Östersund Sweden

**Keywords:** androgen antagonists, biomarker, MMP‐8, periodontal diseases, saliva

## Abstract

**Introduction:**

Matrix metalloproteinase‐8 (MMP‐8) is considered as one of the most promising diagnostic markers for periodontal disease. Androgen deprivation therapy (ADT) has been correlated with impaired collagen synthesis and an increase in periodontal tissue susceptibility to pathogenic microorganisms.

**Objective:**

This study aims to investigate the impact of ADT on salivary MMP‐8 level and periodontal parameters, which might be useful in monitoring periodontal disease in prostate cancer patients undergoing ADT.

**Materials and Methods:**

A total of 88 subjects were selected and were divided into two groups: Group I included *n* = 78 PC patients who have been undergoing ADT); Group II included *n* = 10 healthy individuals. Periodontal parameters such as plaque index (PI), gingival index (GI), periodontal probing depth (PPD), and clinical attachment level (CAL) were examined. The salivary MMP‐8 level was estimated by using the sandwich enzyme‐linked immunosorbent assay method.

**Results:**

Significant differences in mean salivary MMP‐8 level were found between PC patients undergoing ADT and healthy individuals. Salivary MMP‐8 levels of all individuals were positively correlated with GI, PI, PPD, and CAL. Salivary MMP‐8 can distinguish between periodontitis and healthy individuals with an accuracy of about 80%.

**Conclusion:**

Salivary MMP‐8 levels were found to be higher in prostate cancer patients undergoing ADT compared to healthy individuals.

## 1 INTRODUCTION

Prostate cancer (PC) is the second most frequent malignant diagnosis (after lung cancer) in men globally which leads to 375,000 deaths (3.8% of all deaths caused by cancer in men) and 1.4 million new cases in 2020 (Sung et al., 2021). Because PC is hormone sensitive, androgen deprivation therapy (ADT) is a primary standard treatment for patients. Despite ADT having a key role in the management of PC, it may cause bone loss in men (Ross & Small, 2002). Substantial evidence suggested that the degradation of periodontal tissues is influenced by systemic bone loss, which is a considerable risk factor for osteoporosis in men (Rees, 2003). The rates of bone loss can be 2%–8% in the lumbar spine region and 1.8%–6.5% in the femoral neck during the initial 12 months of continuous ADT (Higano, 2003; Seeman & Eisman, 2004; Wei et al., 1999).

Matrix metalloproteinases (MMPs), in particular, play a significant role in the regulation of connective tissue degradation in periodontal disease. MMP expression is significantly low under normal physiological conditions, and this low level is associated with healthy connective tissue remodeling (Checchi et al., 2020). MMP expression increases significantly under pathologic conditions such as periodontal disease, resulting in abnormal connective tissue breakdown, and thus, an increase in MMP levels in saliva (Sapna et al., 2014). MMPs consist of a family of enzymes that cleave protein substrates through a homologous mechanism that involves the activation of an active site‐bound water molecule by a Zn^2+^ ion that is involved in the physiological breakdown of extracellular matrix protein and basement membrane. MMPs are classified into various groups: collagenase (MMP‐1, ‐8, ‐13), gelatinase (MMP‐2, ‐9), stromelysin (MMP‐3, ‐10, ‐11), matrilysins (MMP‐7), and other MMPs related to membranes (Laronha & Caldeira, 2020). MMP‐8 is a collagenase that has the distinct potential to disintegrate the collagen mainly in Type I and Type III (Patricia et al., 2017). As a result, MMP‐8 has been identified as a significant biomarker in periodontal disease (Zhang et al., 2018). It plays a crucial role in the breakdown of a collagen metalloproteinase that is predominantly found in local gingival tissues and oral fluids, and its concentration is associated with periodontal inflammation, as well as bleeding on probing, probing pocket depth, and attachment loss (Giannobile et al., 2009; Kraft‐Neumärker et al., 2012). A study conducted in 2007 by Famili P et al. demonstrated that the prevalence of periodontal disease was significantly higher in a group with PC undergoing ADT compared with the group with PC who were not undergoing ADT (Famili et al., 2007). However, the periodontal examination of this study only included the assessment of plaque index (PI), gingival index (GI), periodontal probing depth (PPD), and clinical attachment level (CAL). Traditional diagnostic techniques such as PPD and CAL have limitations in the present era of oral medicine and therapeutics. To overcome these limitations, our study adopted a comprehensive periodontal examination including the assessment of PI, GI, PPD, and CAL, as well as the evaluation of the level of salivary MMP‐8, a potential diagnostic biomarker of periodontal disease, in prostate cancer patients undergoing ADT compared with the healthy individuals. This study aims to investigate the impact of ADT on salivary MMP‐8 level and periodontal parameters, which might be useful in monitoring periodontal disease in prostate cancer patients undergoing ADT.

## 2 MATERIALS AND METHODS

### 2.1 Patient selection

All participants were recruited at the Institute of Pathology and Diagnostic Medicine, Khyber Medical University, Peshawar, Pakistan from the Institute of Kidney Diseases, Peshawar, Pakistan, between September 2021 and February 2022. All participants were individually informed about the purposes of the study, and written informed consent was obtained. This cross‐sectional study was approved by the Institutional Review Board of Khyber Medical University, Peshawar, Pakistan. The study was conducted in full accordance with the Declaration of Helsinki.

Our study targeted prostate cancer patients undergoing ADT for at least 3 months for Group I. For Group II healthy individuals (prostate cancer‐free).

Individuals diagnosed with metastatic prostate cancer, age less than 50 years, smoker, any comorbidity, have been taking chemotherapy and radiotherapy, and those who have less than 20 natural teeth were excluded.

The sample size was determined using the following formulation for obtaining the specificity and sensitivity according to the World Health Organization “Health Studies Sample Size Definition” (Serdar et al., 2021). A total of 96 participants were recruited, among which eight participants were excluded due to comorbidity. The remaining 88 participants were in the age group 50 to 80 and had at least 20 natural teeth been assigned into two groups: Group I (*n* = 78): Prostate cancer patients undergoing ADT for at least 3 months, and Group II (*n* = 10): healthy individuals.

### 2.2 Clinical history proforma

The principal investigator filled out a clinical history proforma with information regarding the demographic profile, including their educational, occupational level, ADT status, and the latest prostate‐specific antigen (PSA) level.

### 2.3 Unstimulated saliva sample collection

Subjects were instructed not to eat or drink for 2 h before the saliva collection. Saliva was collected between 8:00 a.m. and 11:00 a.m. They were instructed to be seated comfortably, in an upright position, and then asked to rinse before 3 ml of unstimulated whole expectorated saliva was collected and deposited into a sterile 50 ml preweight sealed sterilized polystyrene container according to the method described by Navazesh (1993). The salivary samples were put immediately on ice. To eliminate cell debris, the saliva was centrifuged (1000*g* for 10 min at 4°C). The sample was then separated from the supernatant and kept in Eppendorf tubes at −80°C until analysis.

### 2.4 Clinical workup

The principal investigator examined all remaining teeth in each subject, including PPD, CAL, bleeding on probing by GI, and PI. All individuals were clinically assessed and had at least 20 teeth. Probing depth was measured from the gingival edge using a calibrated Naber's Probe with a 0.5 mm diameter. To assess probing depth, six sites per tooth were probed (distobuccal, mid‐buccal, mesiobuccal, distolingual, mid‐lingual, and mesiolingual). The clinical attachment loss is assessed in millimeters from the cementoenamel junction to the base of the periodontal pocket. Periodontal disease was defined as attachment loss of 2 mm or more in more than two interproximal sites (not on the same tooth). The probing depth is the distance between the free gingival margin and the base of the sulcus/pocket that can be probed. A participant was considered to have periodontal disease if his probing depth was 4 mm or more in more than two interproximal areas (not on the same tooth). Gingival bleeding was graded as 0—no bleeding on probing and 1—bleeding on probing. The supragingival plaque was graded as 0—no plaque and 1—plaque. Periodontal disease was assumed if any of these signs were present. A standard dental form was utilized.

### 2.5 Enzyme‐linked immunosorbent assay (ELISA) analysis of MMP‐8

To detect salivary MMP‐8 concentration in the study participant, the researchers used a commercially available ELISA Kit named Elabscience® (Human MMP‐8 ELISA Kit, Catalog No.: E‐EL‐H1450, 14780 Memorial Drive, Suite 216, Houston, Texas 77079, USA). This ELISA Kit includes optimized reagents that are ready to use as per the manufacturer's instructions. Absorbance was read at 450 nm. The minimum detectable dose of MMP‐8 ranged from 132.30 to 428.20 ng/ml in the current study.

### 2.6 Statistical analysis

Continuous data were presented as mean ± standard deviation while discrete (categorical) in percentage. The data were analyzed using the Statistical Packages for Social Sciences (SPSS) version 22. An independent *t* test was used to compare PSA value and MMP‐8 concentration between groups.

Correlations between MMP‐8 and periodontal parameters were identified using Spearman's rank correlation coefficients. The confidence level was set at *p* < .05.

The diagnostic potential of a biomarker was assessed by a receiver operating curve (ROC) and calculated the area under the curve (AUC). For each point on the ROC curve, the sensitivity and specificity were determined. The cut‐off value of MMP‐8 was also determined.

## 3 RESULTS

### 3.1 Demographic analysis

A total of 96 participants were recruited, among which eight participants were excluded due to comorbidity (edentulous: *n* = 4; diabetes: *n* = 3; hypertension: *n*= 1). The remaining 88 participants were in the age group 50 to 80 and had at least 20 natural teeth assigned to Group I (*n* = 78): Prostate cancer patients undergoing ADT for at least 3 months, and Group II (*n* = 10): healthy individuals.

The mean ± standard deviation (SD) age of Group I was 65.3 ± 7.086 years with a range of 53–81 years.

The level of education in Group I was divided into the following six groups: 19.2% had not attained education, 7.7% were under primary education, 11.5% were in primary education, 7.7% were in middle education, 11.5% were in secondary education, 19.2% had attained matriculation, 2.6% had attained higher secondary education, 16.7% had attained graduation, and 3% had attained masters education.

In Group I, 10.3% were retired or government employees, 21.8% were private employees, and 67.9 were earning at home or doing business.

The mean ± SD of Group II was 62± 3.3 years with an age range of 56–68 years.

The level of education in Group II was 20% had not attained education, 10% were in primary education, 20% were in secondary education, and 50% had attained graduation.

In Group II 40% were on private jobs and 60% were earning at home or doing business. None of the subjects (case) were government employees.

Demographic characteristics of Group I and Group II including their occupation and level of education are shown in Table 1.

**Table 1:** Demographic factors of Group I and Group II

		Percentage (%)	
Demographic characteristics	Category	Group I (*n* = 78)	Group II (*n* = 10)
Occupation	Government job	10.3	0
	Private job	21.8	40
	Business/home	67.9	60
	Total	100	100
Level of education	No education	19.2	20
	Under primary	7.7	0
	Primary	11.5	10
	Middle	7.7	0
	Secondary	11.5	0
	Matric	19.2	0
	Higher secondary	2.6	20
	Graduate	16.7	50
	Masters	3.8	0
	Total	100	100

### 3.2 PSA level

The mean ± SD PSA level of Group I was 7.92 ± 8.81 ng/ml with a range from 1 to 50.5 ng/ml, and in Group II it was 2.05 ± 0.59 ng/ml with a range of 1.50–2.50 ng/ml. The differences between groups were significant (*t* = 2.096 *p* < .05).

### 3.3 Salivary MMP‐8

The mean ± SD concentration of MMP‐8 in Group I (250.74 ± 50.69 ng/ml) was significantly higher than in Group II (176.48 ± 20.41 ng/ml) (*t* = 4.566 *p* < .05).

### 3.4 Correlations between PI, GI, CAL, PPD, and salivary MMP‐8

There was a weak to moderate positive correlation (Dancey & Reidy, 2007) between clinical periodontal parameters and salivary MMP‐8.

PI showed moderate significant positive correlations with the concentration of salivary MMP‐8, while GI, CAL, and PPD showed a weak significant positive correlation with the concentration of salivary MMP‐8 as shown in Table 2.

**Table 2:** Spearman's rank correlations between MMP‐8 and clinical periodontal parameters

Parameters	Spearman's *ρ* (concerning MMP‐8)	*p* Value
Gingival index	0.299	.005^a^
Plaque index	0.400	.001^a^
Clinical attachment loss	0.340	.000^a^
Periodontal probing depth	0.343	.001^a^

Abbreviation: MMP, matrix metalloproteinase‐8.

^a^ Correlation is significant at the .01 level (two‐tailed).

### 3.5 Diagnostic potential of salivary MMP‐8

Receiver‐operating characteristic (ROC) curves generally employed the technique of assessing diagnostic testing effectiveness. The curve depicts sensitivity versus specificity (true positive rate or 1 − specificity), respectively, and can vary throughout studies.

The closer a ROC curve is to the top left corner, the more efficient the diagnostic test (Zweig & Campbell, 1993). The higher the AUC value, the better the accuracy of the diagnostic test.

**Figure 1:**
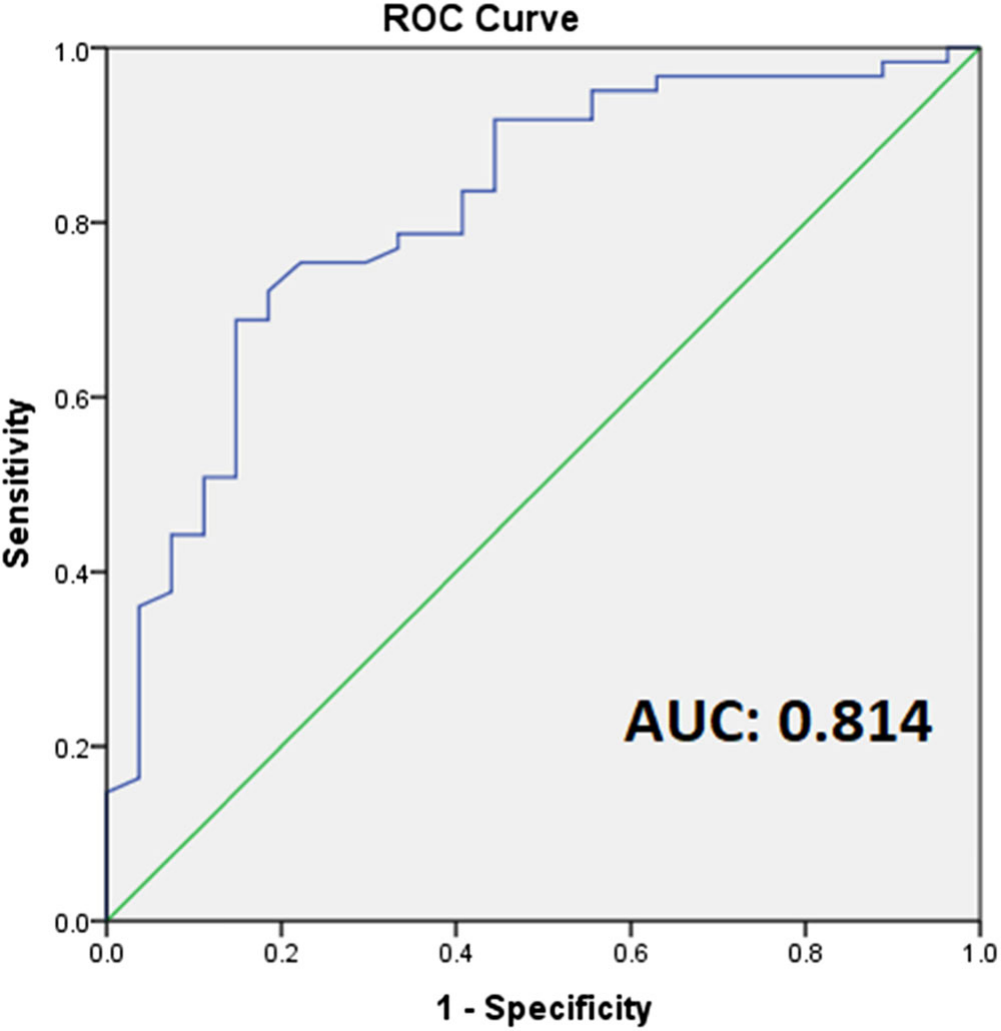
ROC analysis of the salivary concentration of MMP‐8 on periodontitis. AUC, area under the curve; MMP‐8, matrix metalloproteinase‐8; ROC, receiver‐operating characteristics.

The specificity and sensitivity of salivary MMP‐8 concentration as a prognostic test were evaluated. The periodontal disease diagnosis of the participants was first divided into binary states of variables with periodontitis positive and periodontitis negative. ROC curve was plotted as shown in Figure 1.

The AUC was 0.814. It is suggested that salivary MMP‐8 can distinguish between periodontitis and healthy individuals with an accuracy of about 80%. This showed that a salivary MMP‐8 level at 214.40 ng/ml or above is an approximate cut‐off value, which indicates periodontitis.

## 4 DISCUSSION

MMP‐8 is a potential biomarker of inflammation found in the oral fluid, which is released from neutrophils. This enzyme has the ability to degrade Type I and Type III collagen, which is vital during periodontal destruction in periodontal disease. Consequently, an increase in MMP‐8 levels is the result of periodontal disease. Several studies found a linear relationship between MMP‐8 levels and periodontal disease severity (Gupta et al., 2015; Salminen et al., 2014; Yuan et al., 2018). Miller et al. (2006) discovered that salivary MMP‐8 levels increase with the severity of the periodontal disease and the MMP‐8 levels were almost 10 times higher in periodontal disease patients than in periodontally healthy individuals. Our study found that MMP‐8 levels were significantly higher among PC patients undergoing ADT who may have lower polymorphonuclear leukocyte chemotaxis, higher levels of collagenase and elastase, and lower amount of collagen synthesis by fibroblasts, all of which contribute to periodontal degradation (Yucel‐Lindberg & Båge, 2013).

Various MMP‐8 measurement techniques with varying degrees of agreement may limit its applicability as an adjunctive tool for periodontal disease screening. According to the current literature, immunofluorometric assay (IFMA) was utilized to assess active MMP‐8 and can be employed as a staging and grading biomarker in the new classification system of periodontitis (Sorsa et al., 2020). In contrast, in the present study, we determined whether or not individuals have periodontal disease using the ELISA technique, which assessed the total MMP‐8. Recently, a study was conducted to assess the ability of MMP‐8 biosensor, IFMA, and ELISA immunoassays to differentiate between periodontal health, gingivitis, and periodontitis. The biosensor data correlated more strongly with total MMP‐8 ELISA data, but less so with active MMP‐8 IFMA, possibly because most of the MMP‐8 in the saliva is in the total and latent forms (Umeizudike et al., 2022).

The majority of previous studies utilized pooled concentrations of gingival crevicular fluid (GCF) from all regions of the mouth to offer an overall assessment of the clinical condition and severity (Leppilahti et al., 2014; Yuan et al., 2018). GCF sample collection in these studies entailed collecting a minimal amount of fluid on filter paper strips, which requires a longer sampling period. Furthermore, contamination of blood and plaque products is common (Buduneli, 2020). Among oral fluids, saliva and mouth rinse can be collected in an uncomplicated, noninvasive, minimal time‐consuming technique by personnel with minimal training. Saliva represents an individual's overall health and aids in the detection of disease at an early stage. Unstimulated saliva was utilized in our study. Since ELISA analysis typically requires <100 μl of the sample, saliva volumes collected during 1–5 min without stimulation were more than adequate for the majority of salivary biomarker analyses. Methods for saliva sample collection to determine salivary biomarker status at rest or in response to a stress stimulus should avoid stimulating saliva flow with a mouth rinse technique or other commonly used stimuli, such as chewing, sour or bitter tastes, and orofacial movements, unless specific rationale and justification warrant such stimuli. The mouth rinse technique causes an increase in sample volume, which increases the sample's water content and consequently dilutes the biomarker concentrations (Gill et al., 2016; Padilla et al., 2020).

ROC curve is a graphical representation of sensitivity against 1 − specificity, which is one of the most common and effective approaches for assessing a diagnostic model's discriminating capacity. Graphical representation of a ROC curve involves plotting various points rather than taking a single estimation, which provides the advantage of the AUC being independent of any specific reference value used in a diagnosis and therefore suitable for analyzing and comparing the effectiveness of distinct biomarkers in various units (Kamarudin et al., 2017). In this study, the ROC curve demonstrated that the level of salivary MMP‐8 could distinguish between individuals with and without periodontal disease with an AUC value of about 0.8. This was in agreement with some studies which found the AUC between 0.8 and 0.9 (Ebersole et al., 2015; Hong et al., 2020; Ramseier et al., 2009).

The primary justification for including healthy participants of the same age group as controls were to set a cut‐off for salivary MMP‐8 concentration. In our study, the concentration/approximate threshold is 214.40 ng/ml, above which there is a possibility of periodontal disease. The cut‐off value of the salivary MMP‐8 level varied considerably between different studies (Hernández et al., 2021; Johnson et al., 2016; Meschiari et al., 2013). This may be due to variations in salivary flow rate, dilution method, antimicrobial drug usage, and smoking habits. These characteristics may interfere with salivary analysis to some degree. However, different detection techniques including ELISA and IFMA may potentially contribute to the variability. Furthermore, the contrasting cut‐off values of salivary MMP‐8 levels between different studies might be attributed to genetic and ethnic variances of the different populations.

ADT has been found to induce considerable bone loss, and that might be the underlying mechanism in the link between periodontal disease and ADT. Our findings corroborate those of a study conducted by Famili et al. (2007), who reported a positive association between prostate cancer patients undergoing ADT and periodontal disease, a greater risk of CAL, and alveolar bone loss.

Our study is the first study, to the best of our knowledge, in which the concentration of salivary MMP‐8 level was analyzed in prostate cancer patients undergoing ADT. The finding of this study further verifies the effect of ADT on periodontal diseases and strengthens the relationship between clinical periodontal parameters and the salivary MMP‐8 level. A limitation of our study pertains to the cross‐sectional study design, which could not infer a causal relationship between periodontal disease severity and the level of MMP‐8. Moreover, it is unknown if the periodontal disease existed before undertaking ADT by PC patients. This cross‐sectional study, however, provides significant preliminary data and sets the framework for future follow‐up investigations.

## 5 CONCLUSION

The concentration of salivary MMP‐8 levels was higher in prostate cancer patients undergoing ADT compared to healthy individuals. Owing to the rising number of prostate cancer diagnosed annually and the increased usage of ADT in the treatment of prostate cancer, this result might have public health implications. Physicians who treat males on ADT ought to be cognizant of the increased need for regular and consistent dental check‐ups to diagnose the periodontal disease early and to prevent tooth loss.

## AUTHOR CONTRIBUTIONS

**Maaz A. Memon**: Conceptualization; data curation; formal analysis; methodology; writing—original draft; writing—review and editing. **Benish Aleem**: Conceptualization; data curation; formal analysis; methodology; writing—original draft; writing—review and editing. **Ka Y. Lee**: Conceptualization; data curation; formal analysis; methodology; writing—original draft; writing—review and editing. **Hifza A. Memon**: Conceptualization; data curation; formal analysis; methodology; writing—original draft; writing—review and editing.

## CONFLICT OF INTEREST

The authors declare no conflict of interest.

## Data Availability

Data are available from the first author upon reasonable request.
